# A Method for Improving the Prediction of Outpatient Visits for Hospital Management: Bayesian Autoregressive Analysis

**DOI:** 10.1155/2022/4718157

**Published:** 2022-10-12

**Authors:** Yanling Wei, Wen Li, Jiyong Tan, Jianhui Yuan, Zhihui Wu, Yu Li, Yu'ang Mao, Daizheng Huang

**Affiliations:** ^1^School of Information and Management, Guangxi Medical University, Nanning, 530021 Guangxi, China; ^2^Department of Biomedical Engineering, Guangxi Medical University, Nanning, 530021 Guangxi, China; ^3^The Laboratory of Biomedical Photonics and Engineering, Guangxi Medical University, Nanning 530021, China

## Abstract

The number of outpatient visits is generally influenced by various factors that are difficult to quantify and obtain, resulting in some irregular fluctuations. The traditional statistical methodology seldom considers these uncertainties. Accordingly, this paper presents a Bayesian autoregressive (AR) analysis to propose a forecasting framework to cope with the strict requirements. The AR model was conducted to identify the linear and autocorrelation relationships of historical series, and Bayesian inference was used to correct and optimize the AR model parameters. Posterior distribution of parameters was stably and reliably obtained by Gibbs sampling on the condition of the convergent Markov chain. Meanwhile, the lag orders of the AR model were adjusted based on the series characteristics. To increase the variability and generality of the dataset, the developed Bayesian AR model was evaluated at seven hospitals in China. The results demonstrated that the Bayesian AR model had varying degrees of decline in the MAPE value in the seven sets of experimental data. The reductions ranged from 0.1431% to 0.0342%, indicating effective optimization of the Bayesian inference in the AR model parameters and reflecting the useful correction of the lag order adjustment strategy. The proposed Bayesian AR framework showed high accuracy index and stable prediction accuracy, thereby outperforming the traditional AR model.

## 1. Introduction

The continuous population growth has increased the demand for medical services in China. Moreover, given the lack of the corresponding growth rate of hospitals, the difficulty in getting medical service has become increasingly serious in recent years. A system for forecasting outpatient visits can be used as a decision support system to improve the outpatient service and patient satisfaction. Therefore, novel methods should be developed to more efficiently forecast outpatient visits and make reasonable decisions for health resource management.

In recent years, the popularity of research by establishing mathematical models of outpatient visits to realize precise forecasting has significantly increased, especially in China. Given that the data of outpatient visits are normally provided in daily or monthly statistics, time series techniques have been primarily exploited to predict outcomes. Compared to univariate forecasting methods, numerous studies have shown that multivariable models improved the predictive performance in many complex system predictive issues [1–3]. Many forecasts have been constructed based on the combination of multiple predictors, which have resulted in a considerable increase in performance with the introduction of machine learning methods. Junfeng et al. employed machine learning methods to predict the peak arrivals of patients with chronic respiratory diseases based on weather and air quality [4].Won et al. employed a multilevel prediction model to predict conjunctivitis in outpatients [5]. Wang et al. created a deep learning model with air pollution concentrations and meteorological variables as predictors to estimate the outpatient visits for cardiopulmonary illness [6]. Most researchers who used multivariate models have mainly focused on some specific diseases and have achieved good results. However, foreseeing the total number of outpatient visits is also very significant, especially for hospital management and operation. More possible unquantifiable confounding factors in outpatient prediction studies, such as personal experience, policy factors, and various environmental factors, may exist. Consequently, forecasting the outpatient visits exactly by multivariate prediction in practice is hard, but applying unitary historical data is more advantageous for forecasting outpatient visits. This method involves the formulation of inferences from single historical data, in which the linear time series model led by the autoregressive integrated moving average (ARIMA) is the most commonly utilized. Lee et al. adopted a two-dimensional hierarchical decision tree scheme to forecast weekly influenza outpatient visits [7]. Deng et al. used backpropagation neural networks to optimize the model hybridized with ARIMA and LSTM model to calculate outpatient visits [8]. Prashanthi et al. created a SARIMA model to estimate the ophthalmology outpatient visits using historical data from the electronic medical record system [9]. The overwhelming majority of research has been implemented using statistical models, which capture information from historical data and quantify the relationship between outpatient visits and explanatory variables or lagged time series to generate forecasts. These methods provide an efficient way to predict outpatient visits by minimizing the data request. However, without the analysis and explanation of mixed interfering factors, uncertainty is inevitable in forecasting outpatient visits. Although the technique described above effectively captures the variation in the data, the potential influence of any other elements has not been captured. The uncertainty of outpatient visits has been handled in a targeting way in a previous research. To make the estimation close to the actual situation, the targeted formulation of inference based on uncertain information is therefore a key strategy to solve the above issues. The authors considered whether some methods could be hybridized to further optimize the parameter estimation of the regression model and sequentially improve the prediction. In this case, Bayesian approach may be a suitable choice. In quantification of uncertainty, the Bayesian approach has been considered to be more flexible than the frequentist approach in exploring the parameter space [10]. In addition, this approach can be integrated with various algorithms due to its good extensibility. Contrary to classical statistical methods, Bayesian methods have two characteristics, namely, the use of prior distributions and the treatment of parameters as unknown random variables [11]. Statisticians have a prior belief on the unknown parameters, which are updated with the observed data and then summarized in the posterior distribution. The flexible hypotheses provide Bayesian methods with high predictive accuracy, making such methods as the preferred method for some areas [12]. Many reports on the application of Bayesian approach in various fields, such as hydrology [13], economics [14], clinic [15], management science [16], and electricity [17], have been published. However, the Bayesian method shows some limitations in practical applications. A large number of unknown parameters should be estimated by using either maximum a posteriori or maximum likelihood to train a model from the data. However, the implementation of Bayesian methods is limited by the computational complexity of traditional multiple integrals and complex posterior distributions. Hence, numerical integration techniques, such as Markov Chain Monte Carlo (MCMC), are required to solve the problem [18].

However, as a cogent strategy in many areas, in all the studies reviewed here, reports on the application of Bayesian inference to forecast outpatient visits are lacking. Therefore, the present work was performed to develop a linear time series model to estimate the monthly outpatient visits based on the Bayesian approach. Its performance in the outpatient visit time series with different characteristics was explored. In the present study, the design of the outpatient visit prediction system was synthetically considered among the data-related characteristics and uncertainty. The Bayesian inference was utilized in the model to provide automatic correction of parameters. Posterior distributions of parameters were approximately simulated by Gibbs sampling algorithm, which avoided the complex integral calculation problem in solving the complete probability function. Next, the model was validated by evaluating how well the models performed against the outpatient visit time series of multiple hospitals (real data) by verifying the model accuracy. Moreover, the prediction results were considered to be in the form of prediction intervals, which provided more valuable information by quantifying fluctuations in the outpatient visits. Finally, to explore the stability of the prediction accuracy of the Bayesian AR model, the training set divided in different proportions was used to train models and calculate predictions. Decomposition algorithm (seasonal and trend decomposition using LOESS (STL)) was applied on each sequence to identify the underlying patterns of the sequences. The association of the patterns and the stability of prediction accuracy was also determined. Then, the best applicable scenarios for the Bayesian AR model were explored. Based on the Bayesian approach, this article provided a novel concept for forecasting possible lines in future outpatient visits and explored whether this methodology enhanced the prediction accuracy compared with the traditional method. The proposed model was aimed to offer hospital administrations with a novel and powerful reference for decisions in health resource management.

## 2. Methods

### 2.1. Data Sources

The developed Bayesian time series framework was evaluated at seven hospitals in China. All data were derived from the published literature. All data of outpatient visits were obtained from the CNKI (http://www.cnki.net/) website, and data were selected based on the following criteria:

1. Data were derived from hospitals distributed in different Chinese provinces
2. The entire hospital historical data should be included
3. Monthly data recorded continuously should be contained in the historical data of each hospital

**Figure 1:**
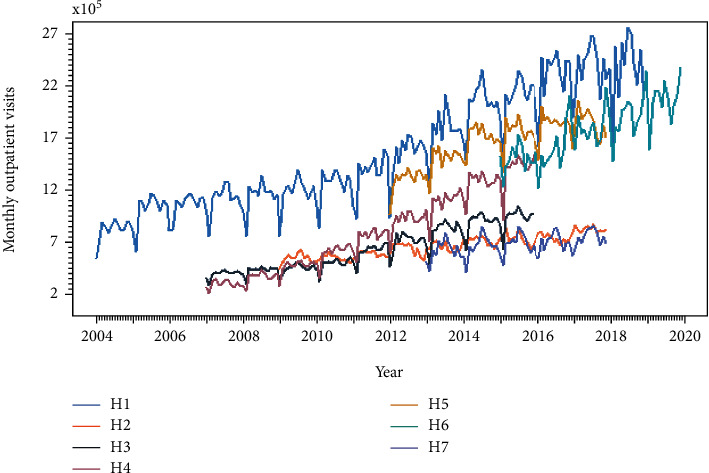
Variation in the outpatient visits with time as collected from the seven hospitals in China.

The data were marked as H1, H2, H3, H4, H5, H6, and H7 as follows: for H1, the first 8 years of data (between 2004 and 2011) from Zhang [19] and the last 7 years (between 2012 and 2018) from Lin et al. [20]; for H2 to H7, data were obtained from Wang et al. [21], Liu [22], Chen et al. [23], Zhou et al. [24], Fu and Gu [25], and Zhou et al. [26]. Data from the seven selected hospitals are briefly summarized in Table 1, and the time sequence is presented in Figure 1. The statistical data, excluding patient personal information, were mainly provided by the Medical Record Information Management System from the hospitals.

### 2.2. Model Establishment

When the historical values display a linear pattern, the classical time series models advantageously capture such structure, especially against nonstationarity data [27]. In the present study, Bayesian autoregressive models, which adopted the Bayesian inference to estimate the parameters, were used.

#### 2.2.1. AR Model

The AR model is suitable for time series with autocorrelation, because the linear combination of previous historical data was used to forecast the future values of interest in it. The AR model is as follows:

(1) $$\begin{array}{c} y_{t}=\alpha +\overset{p}{\underset{t=1}{\sum }}\beta_{k}y_{t-k}+\epsilon_{t}, \end{array}$$

where *p* is the order of lag, *Y*_*t*_ is the current time series value, *Y*_*t*−*k*_ (*k* = 1, 2, ⋯, *p*) represents the past values, *α* is a constant, *β*_*k*_ (*k* = 1, 2, ⋯, *p*) represents autoregressive coefficients, and *ε*_*t*_ is a zero-mean white noise, *ε*_*t*_~*N*(0, *σ*^2^).

In the AR model, finding an appropriate lagged value is the key to a good prediction. In the present study, the Akaike information criterion (AIC) was introduced to resolve the problem. AIC is one of the most common measures for determining the optimum order p of the AR model, and the value of *p* that minimizes the AIC was chosen [28]. AIC is defined as follows:

(2) $$\begin{array}{c} \text{AIC}\left(p\right)=\log\frac{\text{SSE}\left(p\right)}{\text{T}-p}+\frac{\left(T-p\right)+2p}{\left(T-p\right)}, \end{array}$$

where *T* is the length of observation periods (training set) and SSE is the residual sum of squares. A residual is defined as the difference between observed and predicted values.

In addition, parameters were calculated using least square estimation with the ARIMA function of the R software.

#### 2.2.2. Bayesian Inference

The MCMC approach based on Gibbs sampling can generate a multiple random variable, which is very complex to be generated directly when the conditional distribution of each component is known [29]. Gibbs sampling-based methods can be used for iterating samples from a posterior conditional distribution and approximating the joint marginal distribution [30]. For the computational efficiency, Gibbs sampling is computationally remarkably faster than other approaches because of the simpler analytic expressions of the full conditional posterior distributions of the model parameters [31]. Therefore, Gibbs sampling was used to perform the Bayesian estimation and obtain the confidence interval of the AR model parameters. In the present study, the Bayesian AR model was built upon incorporating the AR model and Gibbs sampling approach. The Bayesian method was introduced to optimize the parameters and thus enabled the model to reasonably consider any potential interactions between the current values of outpatient visits and historical data. In general, the AR model can be considered as a linear regression of the following form:

(3) $$\begin{array}{c} y_{t}={\beta x}_{t}+\epsilon_{t}. \end{array}$$

The likelihood function is as follows:

(4) $$\begin{array}{c} F\left(y_{t}\left|\beta ,\sigma^{2}\right.\right)={\left(2\pi \sigma^{2}\right)}^{-T/2}\exp\left[-\frac{{\left(y_{t}-\beta x_{t}\right)}^{\text{T}}\left(y_{t}-\beta x_{t}\right)}{2\sigma^{2}}\right]. \end{array}$$

The formula for the Bayes rule is stated as follows:

(5) $$\begin{array}{c} P\left(A\left|B\right.\right)=\frac{P\left(B\left|A\right.\right)P\left(A\right)}{P\left(B\right)}. \end{array}$$

From the Bayesian theory, the parameters of the AR model are random variables with probability distribution, and the posterior probability of parameters can be written as follows:

(6) $$\begin{array}{c} H\left(\alpha ,\beta ,\sigma^{2}\left|y_{t}\right.\right)=\frac{F\left(y_{t}\left|\alpha ,\beta ,\sigma^{2}\right.\right)P\left(\alpha ,\beta ,\sigma^{2}\right)}{F\left(y\right)}. \end{array}$$

The denominator [*F*(*Y*)] does not depend on the parameters. Therefore, the posterior distribution can be further described as follows:

(7) $$\begin{array}{c} H\left(\alpha ,\beta ,\sigma^{2}\left|y_{t}\right.\right)\propto F\left(y_{t}\left|\alpha ,\beta ,\sigma^{2}\right.\right)P\left(\alpha ,\beta ,\sigma^{2}\right). \end{array}$$

The AR model has a joint distribution of *P* + 2 variables and can written as *f*(*α*, *β*_1_, *β*_2_, ⋯, *β*_*P*_, *σ*^2^). The Gibbs sampling process can be described as follows.

**Step 1 .**

The initial values of all variables should be set.

(8) $$\begin{array}{c} \phi^{\left(0\right)}=\left(\alpha^{\left(0\right)},\beta_{1}^{\left(0\right)},\cdots ,\beta_{P}^{\left(0\right)},{\sigma^{2}}^{\left(0\right)}\right), \end{array}$$

where the numbers in the parentheses (at the top-right of variables) represent the current state of the variables in the Gibbs sampling process.

**Step 2 .**

Each variable was sampled from the conditional distribution in sequence. In this case, *α*^(1)^ was sampled from the current values of the other (*P* + 1) variables described as follows:

(9) $$\begin{array}{c} f\left(\alpha^{\left(1\right)}\left|\beta_{1}^{\left(0\right)},\cdots ,\beta_{P}^{\left(0\right)},\sigma^{2\left(0\right)}\right.\right). \end{array}$$

Then, *α*^(0)^ was replaced with *α*^(1)^ and constituted a new conditional distribution. Then, *β*_1_^(1)^ was sampled conditionally on this new distribution as follows:

(10) $$\begin{array}{c} f\left(\beta_{1}^{\left(1\right)}\left|\alpha^{\left(1\right)},\beta_{2}^{\left(0\right)},\cdots ,\beta_{P}^{\left(0\right)},\sigma^{2\left(0\right)}\right.\right). \end{array}$$

This process was repeated, until each variable had been sampled, and the first iteration of sampling was completed. The initial values of all variables were obtained for the following iteration:

(11) $$\begin{array}{c} \phi^{\left(1\right)}=\left(\alpha^{\left(1\right)},\beta_{1}^{\left(1\right)},\cdots ,\beta_{\text{P}}^{\left(1\right)},{\sigma^{2}}^{\left(1\right)}\right). \end{array}$$

The above step was repeated *M* times, and the sample series was obtained and represented as (*ϕ*^(1)^, *ϕ*^(2)^, ⋯, *ϕ*^(*M*)^). The anterior-*N* groups were discarded, and the remaining sample series converged to a stationary distribution independent of the initial value once *M*‐*N* was large enough, in which the mean values were used as the estimates of each parameter.

Once the posterior parameters were generated based by Gibbs sampling, forecasts of outpatient visits were calculated based on the Bayesian AR model by using the parameters obtained above. In *M* iterations of the Gibbs sampling algorithm, *M* posterior samplings of each parameters were generated. The corresponding *M* forecast values were obtained for each point in the forecast horizon.

For the Gibbs sampling algorithm, iterations equal to 20,000 were set in the Gibbs sampling algorithm, and prior distributions of parameters were set up below:

(12) $$\begin{array}{c} p\left(\alpha \right)\sim N\left(0,1\right),\\ p\left(\beta_{K\left(K=1,\cdots ,\text{p}\right)}\right)\sim N\left(0,1\right),\\ p\left(\varepsilon \right)\sim \Gamma^{-1}\left(\frac{1}{2},\frac{1}{20}\right). \end{array}$$

For *α* and *β*, normal distribution with mean of 0 and variance of 1 was set as prior distributions. For *ε*, inverse gamma distribution with shape parameter of 1/2 and scale parameter of 1/20 was set as prior distribution.

In each iteration of the Gibbs sampling algorithm, the parameters of the *AR*(*P*) model were obtained using the MCMC approach based on Gibbs sampling, in which the predicted values in the forecast horizon were calculated. The effectiveness and stability of Gibbs sampling algorithm can be proved under global convergence. The simulation results of Gibbs sampling could be considered as an acceptable approximation of posterior distribution once the Markov chains converge to a stable state.

**Figure 2:**
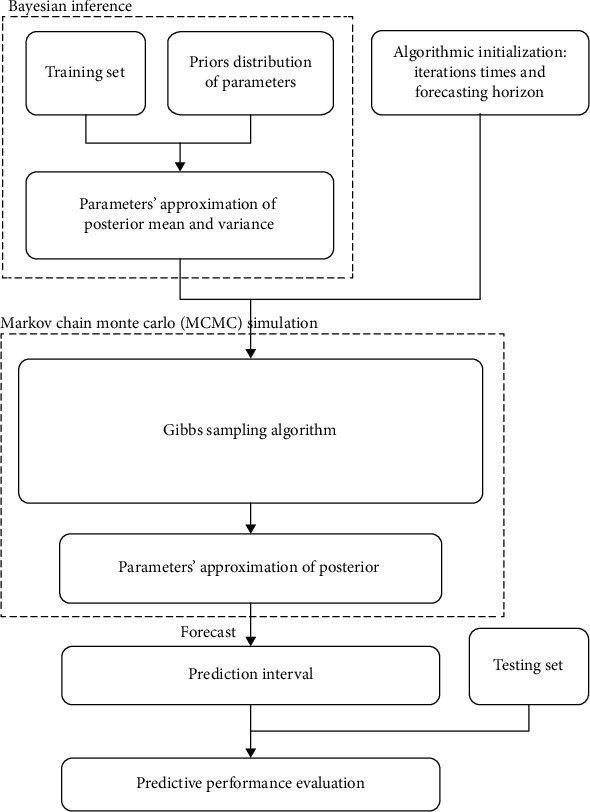
Workflow for the development of the outpatient visit prediction system.

In all models, the anterior 80% of the outpatient visits dataset was used as the training set, and the posterior 20% of the dataset was used as the testing set. The test set was masked during model fitting. The flowchart for the algorithm is presented in Figure 2. The predicted mean was used as a point estimate for each month and the prediction standard deviation as a measure of forecasting uncertainty. The prediction accuracy of each model in the different methods needs to be understood, and error metric indices, including root mean squared error (RMSE) and mean average percent error (MAPE), were adopted to evaluate the performance of the two forecasting frameworks. A good forecasting performance indicates a low RMSE or MAPE, which are defined as follows:

(13) $$\begin{array}{c} \text{RMSE}=\sqrt{\frac{1}{n}\overset{n}{\underset{i=1}{\sum }}{\left(y_{t}-{\overset{\wedge }{y}}_{t}\right)}^{2}}, \end{array}$$

(14) $$\begin{array}{c} \text{MAPE}=\frac{1}{n}\overset{n}{\underset{\text{i}=1}{\sum }}100\frac{\left|{\hat{y}}_{t}-y_{t}\right|}{y_{t}}, \end{array}$$

where ${\hat{y}}_{t}$ is the forecasted value (represented by predict mean), *y*_*t*_ is the corresponding observation, and *n* is the sample size of the testing set.

## 3. Results

**Figure 3:**
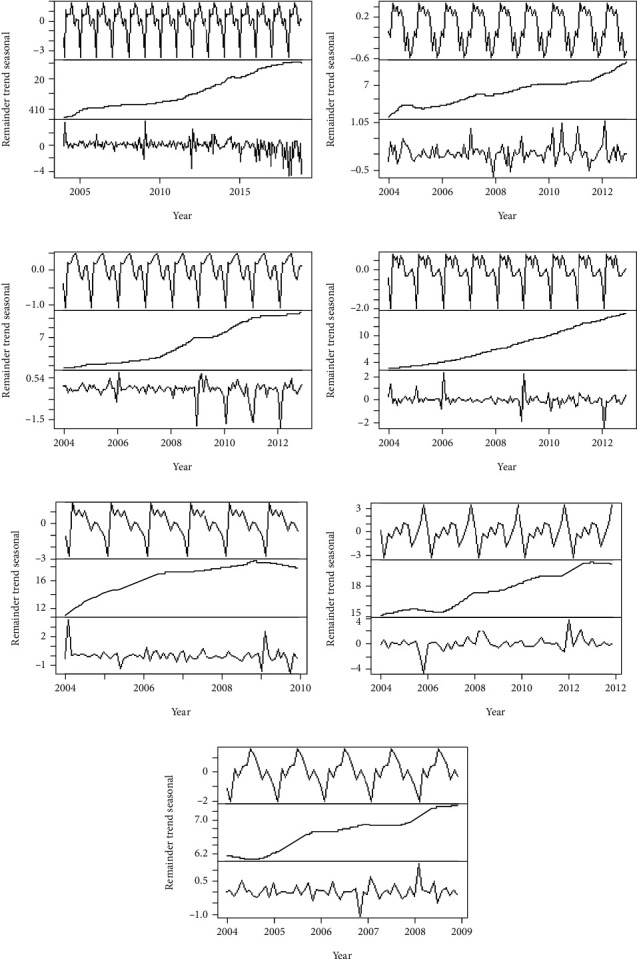
Three additive components obtained from a robust STL decomposition from the seven outpatient visits datasets.

The seven sets of validated data were first comparatively analyzed via descriptive statistics, which included kurtosis, skewness, long-term trend, periodicity, and complexity. The data were first comparatively analyzed via descriptive statistics. The kurtosis and skewness were calculated by the moment package of the R software, and permutation entropy could effectively reflect the complexity of the time series data [32], as shown in Table 2. The periodicity, the long-term trend, and the complexity of the seven series were displayed by adopting the STL decomposition (Figure 3. Each time series was split into three components, namely, seasonal, long trend, and remainder components. The comprehensive analysis of above features showed that all datasets exhibited kurtosis and heavy-tail distributions. In the subcomponents of the STL decomposition in Figure 3, obvious seasonal and long-term trend features were observed in the seven datasets. It can also be observed that the remainder components have different dynamic patterns. This result suggests that there are other patterns besides seasonal and long trend that control outpatient volume accumulation. In Table 2 and Figure 3, the higher complexity could be expressed as a larger value of permutation entropy or some irregular change in the remainder curves.

The models described above were fitted to the monthly outpatient visits for each hospital in the seven locations. First, the AIC values were calculated from 1 to the maximum order of the models, and the optimal orders in each time series were selected, in which the maximum orders were set as 1/2 of the number of observations in the training set. The optimal order was determined by the corresponding *p* value, which minimized the AIC value, based on which the models were built. For the Bayesian approach, once the posterior sampling of parameters was generated by Gibbs sampling, the Bayesian AR models, in which the parameters were defined as the sampling results, were used to calculate outpatient visits. After the first 4,000 iterations were discarded as burn-in, 1,600 forecast values were obtained for each month (within the forecast horizon), which constituted the prediction intervals. To show the superiority of the Bayesian AR forecasting model, the AR model was adopted as the baseline based on the traditional method. The forecasting errors in the outpatient visit by using the AR model and the Bayesian AR forecasting model at the selected locations are summarized in Table 3. For the RMSE and MAPE, the Bayesian approach had substantially lower values than those with of traditional approach, especially the evident decline in MAPE by over 0.10% in H3 and H4. Overall, the accuracies of the Bayesian AR forecasts were better than those of the traditional AR forecasts. In addition, data from the same hospital selected had different orders for the two models, which were attributed to the AIC results depending on the model residual. In theory, as a generic methodology of order determined for the AR model, minimized AIC selects an appropriate order for the two models based on the same dataset and penalty.

**Figure 4:**
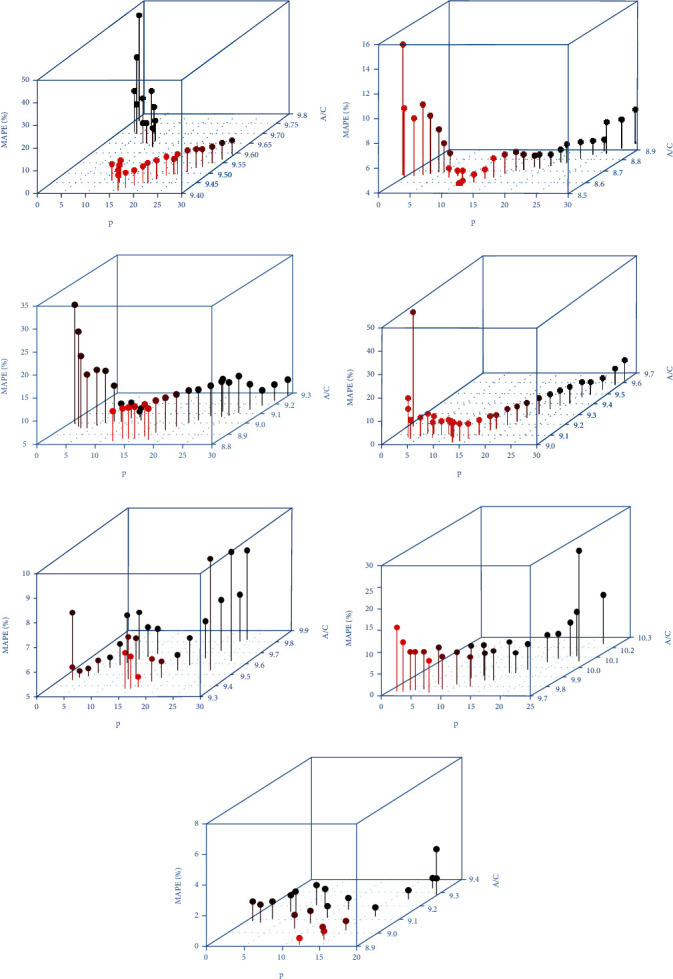
Comparison of the AICs and MAPEs of the different models in increasing order at the different hospitals.

Based on Table 3, the lag order selected was generally low. However, based on the expressions of the AR model, very limited historical data are likely to result in the reduction of the calculative MAPE of the model. A large historical data may result in overfitting. In this case, to further explore the optimal performance of the method, the search range of the lag order for the Bayesian AR models was expanded. In this paper, searches of order were restricted to 1/2 of the number of training sets. The ARIMA function of the R software has a required process of checks for stability before fitting the data. The function reported an error while the order of the AR model was raised. The optimal performance of the AR model was not explored further in this study. To determine the appropriate order more intuitively, the AIC values and prediction accuracy of the models were calculated while the *p* value was raised, as presented in Figure 4. Figure 4 displays the 3D scatter diagram of the relationship among the order, AIC, and MAPE results. As the *p* value increased, the AIC values declined gradually, and some values reached a significantly low point. Some values tended to stabilize at *p* values of 12–20 and finally continued to increase. However, in any case of AIC values described above, the best prediction performance of the majority of hospital was achieved at *p* values of 12–16. The *p* value that minimized the AIC value was selected as the order for each hospital and showed the prediction accuracy in Table 4. Comparison of Tables 3 and 4 showed the low RMSE and MAPE values. After modifying the lag orders of the Bayesian AR models, the MAPE values decreased to 0.073%, 0.043%, 0.136%, 0.086%, 0.070%, 0.073%, and 0.067%. These results showed performance benefits.

Once the models were determined, the posterior parameter distributions were obtained based on the Bayesian inference. Models were simulated 20,000 times with parameters sampled from these posterior distributions. With the first 4,000 iterations removed as burn-in, the posterior density provided the shape of the posterior parameter distributions (Figure S1 in the Supplementary Material). Figure S1 shows that all samples were similar in shape to a normal distribution. The trace plots from the Gibbs sampling are presented in Figure S2 in the Supplementary Material. The sampling sequence of each parameter in Figure S2 were concentrated, close to some value, and fluctuated within a narrow range. Hence, the Markov chains formed by the sampling results were convergent and stable, which confirmed the reliability of the parameter estimation process.

**Figure 5:**
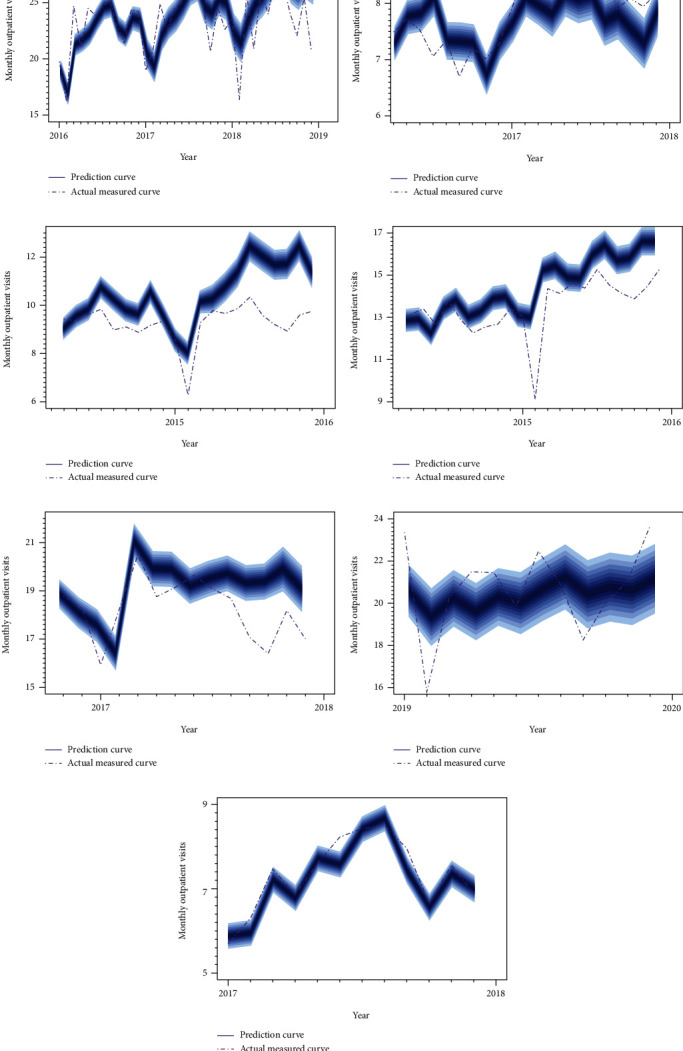
Forecasts in the outpatient visit in seven hospitals based on the Bayesian AR model.

The AR model readily generated multistep predictions on the condition of acquiring parameters. The forecasting horizon was designed to match the length of each testing set. Based on the generated sampled parameter, 16,000 predicted values were obtained for each month, comprising the predicted distribution. Means and variances were calculated using these predicted values. To accurately visualize the forecasts, the 90 quantiles were converted into 17 prediction intervals {*I*_*α*_ = (0.10, 0.15, ⋯, 0.90)} with 5% increment. The blue curves included prediction intervals in each point in Figure 5, which shows the outpatient visits forecasts in the seven hospitals. The graph was generated from the Bayesian AR model. In Figure 5, the prediction curves were roughly closer to the actual measured curves and greatly described the tendency of the outpatient visits. However, some relatively large errors were found toward the end of the prediction horizon compared with the front section, especially in H3, H4, and H5.

**Figure 6:**
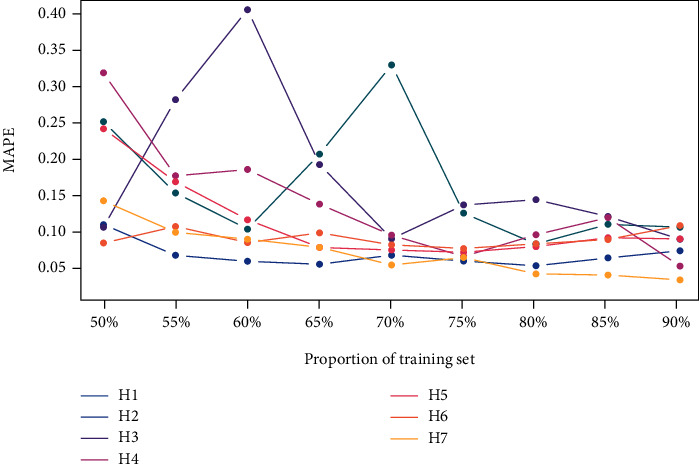
Variation curve of the model accuracy in the seven hospitals.

The stability of the prediction accuracy of the seven hospital models was also examined (Figure 6). To perform an objective evaluation, training sets were divided into the following proportions: 50%, 55%, 60%, 65%, 70%, 75%, 80%, 85%, and 90%. The seven color lines in Figure 6 indicated the MAPE values of the seven hospitals being forecasted in the different training sets. The prediction accuracy maintained superior stability, except for H1 and H3 (the green and the purple lines in Figure 6). MAPE values were typically higher when the percentage of the training sets in the entire sequence was lower (notice the point at 50% in Figure 6) and gradually declined as this proportion increased. When the training set was between 70% and 80% of the total sequence, lower MAPE values (mostly concentrated between 0.03% and 0.08%) were essentially achieved.

## 4. Discussion

As shown in Figure 1, intensity of outpatient volume seasonality remained consistently detectable but with varying intensity throughout the seven datasets we collected. But unfortunately, very little is known about the mechanisms behind the process of outpatient volumes production, especially in the case of public health epidemic. The seven seasonal datasets still tell us little about whether the seasonal pattern is the inherent characteristics of outpatient volume. Therefore, it seems logical that AR, whose forecasts do not depend on seasonal observed data, would be a more appropriate model to ensure more sensitive outpatient visits changes.

Considering the high uncertainty in the number of outpatient visits, the inference can be strengthened substantially by using adequate multivariate statistical and mathematical models. Thus, multivariate time-varying parameter modeling to track patterns in outpatient visits has been suggested. However, such tools require numerous factors, such as the time-varying nature of the underlying ecological and climatic scenarios and the policy and social behavioral influences involved in hospital selection. In actual application, the development of a more complex model is difficult to implement not only because of the challenge of fully understanding both intrinsic and extrinsic complex factors but also the relative scarcity of available data. The current research have mainly focused on relying on univariate trend of the time series data and used traditional time series models to capture patterns. The authors further propose the introduction of Bayesian inference to consider uncertainty associated with parameters.

As mentioned in the literature review, Bayesian inference can effectively express uncertainty. However, no relevant studies on the prediction of outpatient visits were found. This study was performed first to determine whether the Bayesian inference could optimize the traditional prediction time series model for outpatient visits. Table 3 exhibits the superiority of the Bayesian method, but these results are not good enough because of order limitation as shown by stability checking. For MAPE, the most significant improvement was displayed in H3, which was lower by approximately 0.114%, and other parameters also declined to varying degrees. These results may support the hypothesis that the accuracy of predicting outpatient visits could be improved by introducing Bayesian inference. This conclusion is in agreement with those obtained [33] in other areas.

**Figure 7:**
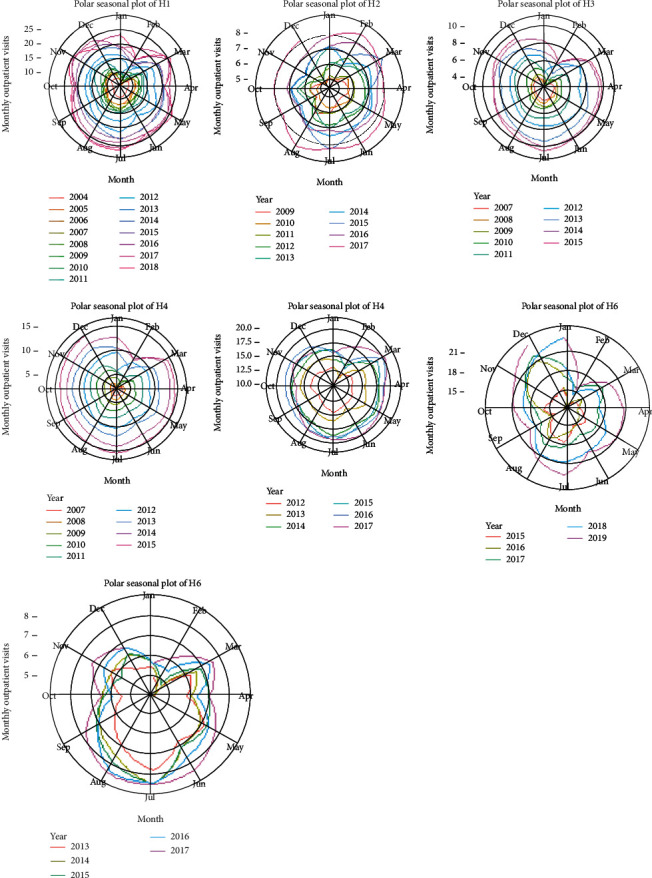
Polar seasonal plot for outpatient visits from the seven selected hospitals.

However, based on the overall RMSE and MAPE results, the results in Table 4 were unsatisfactory. The findings may have been influenced by the lower lag order, because the majority of the determined time lag were only within the range from 1 to 5, except for H5. Moreover, various disease seasonality has been linked to annual climate seasonality as observed by other researchers, such as respiratory infections [34, 35], eye illness [36], and skin disease [37]. Therefore, the number of outpatient visits usually shows seasonal variations.  Figure 7 illustrates the seasonal trends in the number of outpatient visits. Except for H6, all datasets at six selected hospitals showed similar geometries with notable seasonal variation. In Figure 7, the data in the different years are represented as different colors. The farther the data point in each month axis was from the center of the circle, the larger were the outpatient visits. The number of outpatient visits in the seven selected hospitals increased annually and displayed higher volatility, reaching the peak between July and August and troughs during January and February every year. If the lag order was not high enough, the learning algorithms could hardly capture substantial insight from the data. The learning model is commonly assumed to perform better if more historical data are integrated into learning. As data argumentation was mentioned by Sukegawa et al. [38], sufficient data should be prepared for learning general-purpose parameters. This rule is not only limited to deep learning models but also applies to time series models. With insufficient lag order, the proposed model was apparently unlikely to completely explain the data pattern. Therefore, the authors disregarded the checking of the model stability to resolve this issue by increasing the lag order. Notably, based on the results in Figure 4, except for H6, the minimum AIC centered on the lag order between 12 and 16. This finding was obtained possibly, because the data of outpatient visits were seasonal.

In addition, compared with the low lag order case, significant improvements were observed in the prediction accuracy at the majority of selected hospitals, as illustrated by the vertical lines that gradually became shorter in Figure 4. For the stability of prediction accuracy, H1 and H3 (the green and purple lines in Figure 6) exhibited significant volatility, while the others were generally more gradual. Hence, the STL decomposition algorithm was employed to identify the underlying patterns of each outpatient visit dataset, as presented in Figure 3. As seen in Figure 3, obvious variation in the remainder components emerged in H1 (Figure 3(a)) and H3 (Figure 3(c)). The pattern change in H1 happened in 2017 and that of H3 in 2009. These unexpected variations in the outpatient visits had various and complex reasons. This unpredictable data volatility is unrecognizable by AR models, resulting in unstable prediction accuracy in H1 and H3. Both alterations in the data patterns transpired at the tail of the sequence, preventing the model from fully learning the pattern. Any model could not deal with such situations, because of the nonaccess to strict conditions but was acceptable for a satisfactory performance of a univariate prediction model.

As expected, the predicted accuracy was further enhanced to various degrees. These results should help researchers to find novel ideas to further enhance the prediction accuracy of outpatient visits. However, the present study has limitations. First, similar to other univariate models, prediction results might be affected by unmeasured confounding factors. Second, the AR model checks for stability before fitting the data, thus limiting the choice of the more probable appropriate order. In the present paper, to further explore the optimal performance of the method, stability checking was eliminated from the algorithm in the Bayesian AR models during fitting. Finally, several relatively high errors were found near the end of the prediction horizon in H3–H5, most probably due to the inability of the model to fully handle the long-term nonlinear information. As a result, in the authors' next investigation, deep learning models with long-term memory should be incorporated in the study design to improve prediction accuracy.

## 5. Conclusion

This study was performed to develop a Bayesian-based AR model for the efficient prediction of outpatient visits. The AR model was applied to identify autocorrelation and linear relationship of series data, and Bayesian inference was used to accurately correct and optimize the parameters of the AR model. Gibbs sampling was adopted to generate a large number of sampling values, which could be considered as a posterior distribution of parameters. Then, the developed Bayesian AR model was evaluated at seven hospitals, and forecasts of outpatient visits were generated. The following conclusions could be drawn:

1. Comparison of the predicted results of the single conventional and Bayesian-based AR model showed that the Bayesian approach could effectively optimize the parameters, resulting in higher performance in forecasting outpatient visits
2. The Gibbs sampling algorithm provided an effective approximation method for parameter estimation. Due to the convergent Markov chain, the generated sampling results of each parameter were stable and reliable
3. Modifying the lag orders of the AR model according to the series characteristics of the outpatient visits could enhance the fitting ability of AR model, thus providing better accuracy in forecasting outpatient visits
4. The developed framework for predicting outpatient visits is robust for hospitals at different locations with distinct scales

For purposes of the management and distribution of medical resources and the formulation of medical policies, these research findings are worthy as a reference for the future design of study protocols.

## Figures and Tables

**Table 1:** Data of outpatient visits as collected from the seven hospitals in China.

Marked symbol	City	Starting time	Ending time	Month
H1	Nanning	2004/1	2018/12	180
H2	Anqing	2009/1	2017/12	108
H3	Xiamen	2007/1	2015/12	108
H4	Xian	2007/1	2015/12	108
H5	Chongqing	2012/1	2017/12	72
H6	Nanjing	2015/1	2019/12	60
H7	Shanghai	2013/1	2017/12	60

**Table 2:** Descriptive and inferential statistical overview of outpatient visit series in the seven hospitals.

	H1	H2	H3	H4	H5	H6	H7
Kurtosis	1.967	2.147	1.720	1.823	2.683	2.270	3.087
Skewness	0.418	0.186	0.342	0.316	-0.587	0.188	-0.350
Permutation entropy	2.528	2.576	2.520	2.494	2.542	2.426	2.479

**Table 3:** Forecasting results of outpatient visits by using AR and Bayesian AR to check the stability of the models.

Model	Metric	Site						
		H1	H2	H3	H4	H5	H6	H7
AR	Order	4	2	1	2	1	1	1
	RMSE	42504.95	11571.18	26656.66	36917.47	22103.58	38841.64	11461.68
	MAPE	0.156%	0.114%	0.258%	0.229%	0.104%	0.173%	0.156%

Bayesian AR	Order	4	5	4	3	12	5	5
	RMSE	31793.72	7347.76	13984.98	12918.25	15705.38	20254.35	9021.714
	MAPE	0.119%	0.081%	0.144%	0.129%	0.074%	0.082%	0.142%

**Table 4:** Forecasting results of outpatient visits by using Bayesian AR at high lag order.

Metric	Site						
	H1	H2	H3	H4	H5	H6	H7
Order	16	13	12	13	14	7	12
RMSE	21930.83	4450.81	15184.97	9295.21	14912.540	18407.43	3862.077
MAPE	0.073%	0.043%	0.136%	0.086%	0.070%	0.073%	0.067%

## Data Availability

Upon request, the authors can send relevant data to verify the validity of the results presented. Such requests should be sent to the corresponding author.

## Supplementary Materials

Supplementary Materials Figure S1: the posterior parameter distributions. The posterior parameter distributions were obtained based on the Bayesian inference. With the first 4,000 iterations removed as burn-in, the posterior density provided the shape of the posterior parameter distributions. It shows that all samples were similar in shape to a normal distribution. Figure S2: the trace plots from the Gibbs sampling. The sampling sequence of each parameter in FigureS2 were concentrated, close to some value, and fluctuated within a narrow range.
