# Supplementary material for: A Method for Improving the Prediction of Outpatient Visits for Hospital Management: Bayesian Autoregressive Analysis
Source: Comput Math Methods Med. 2022 Oct 12;2022:4718157. doi: 10.1155/2022/4718157 (PMC9581652; doi:10.1155/2022/4718157)
Supplement: Supplementary Materials — Figure S1: the posterior parameter distributions. The posterior parameter distributions were obtained based on the Bayesian inference. With the first 4,000 iterations removed as burn-in, the posterior density provided the shape of the posterior parameter distributions. It shows that all samples were similar in shape to a normal distribution. Figure S2: the trace plots from the Gibbs sampling. The sampling sequence of each parameter in FigureS2 were concentrated, close to some value, and fluctuated within a narrow range. [file 4718157.f1.docx]

**A method for improving the prediction of outpatient visits for hospital management: Bayesian Autoregressive analysis**

SUPPLEMENTARY FIGURES


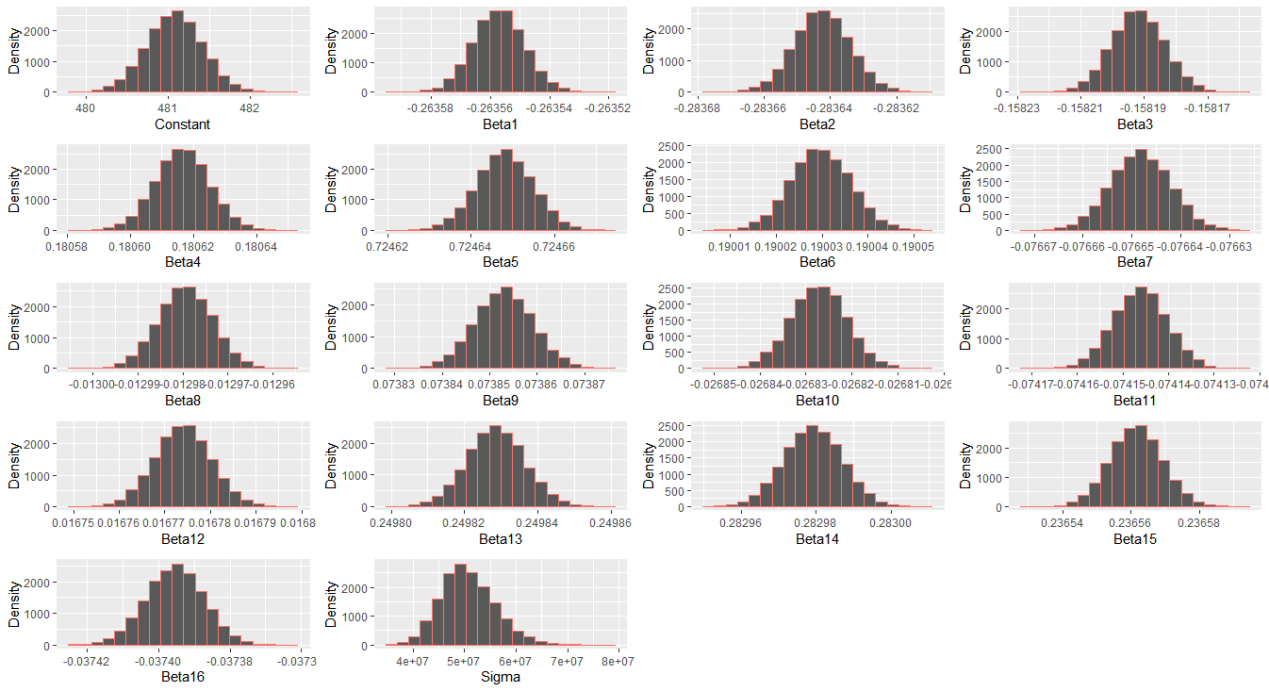


1. H1


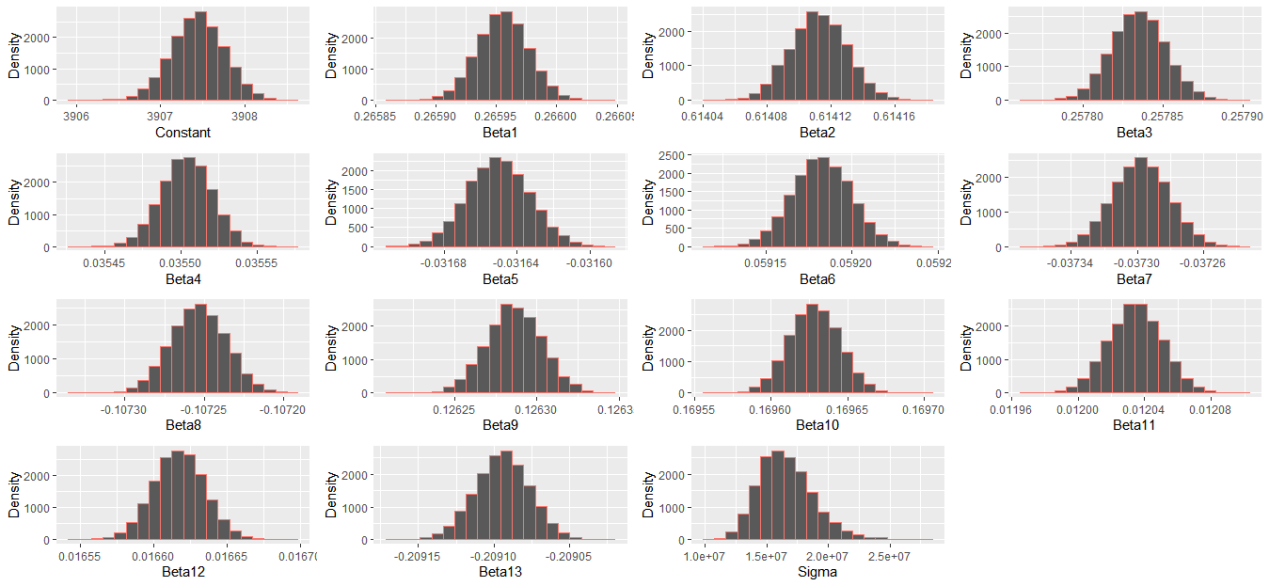


1. H2


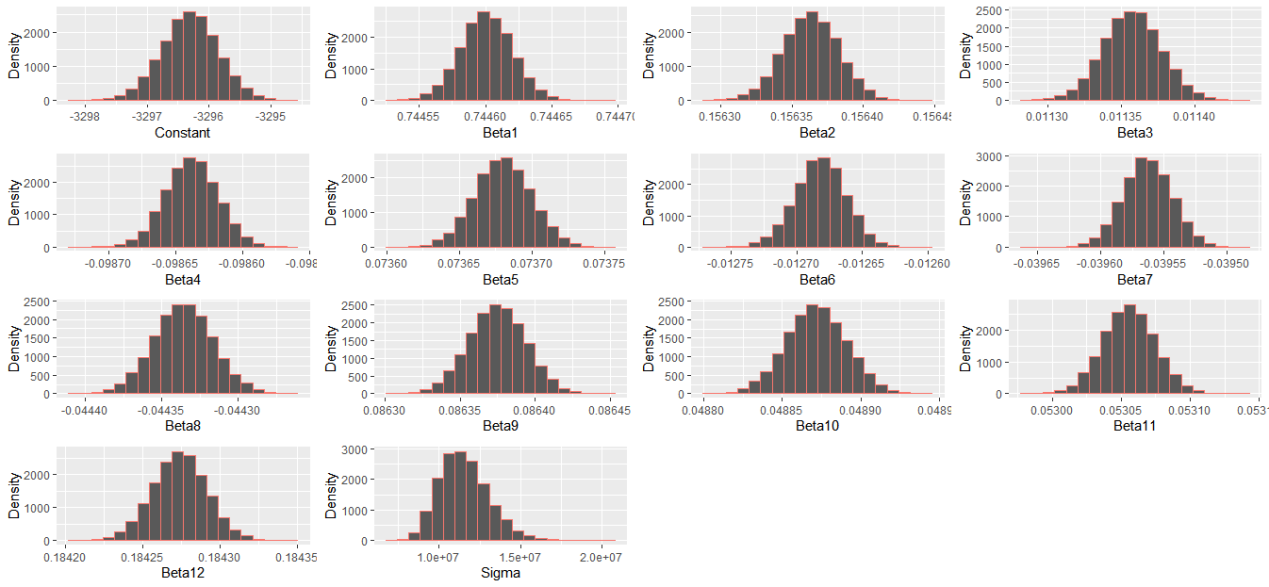


1. H3


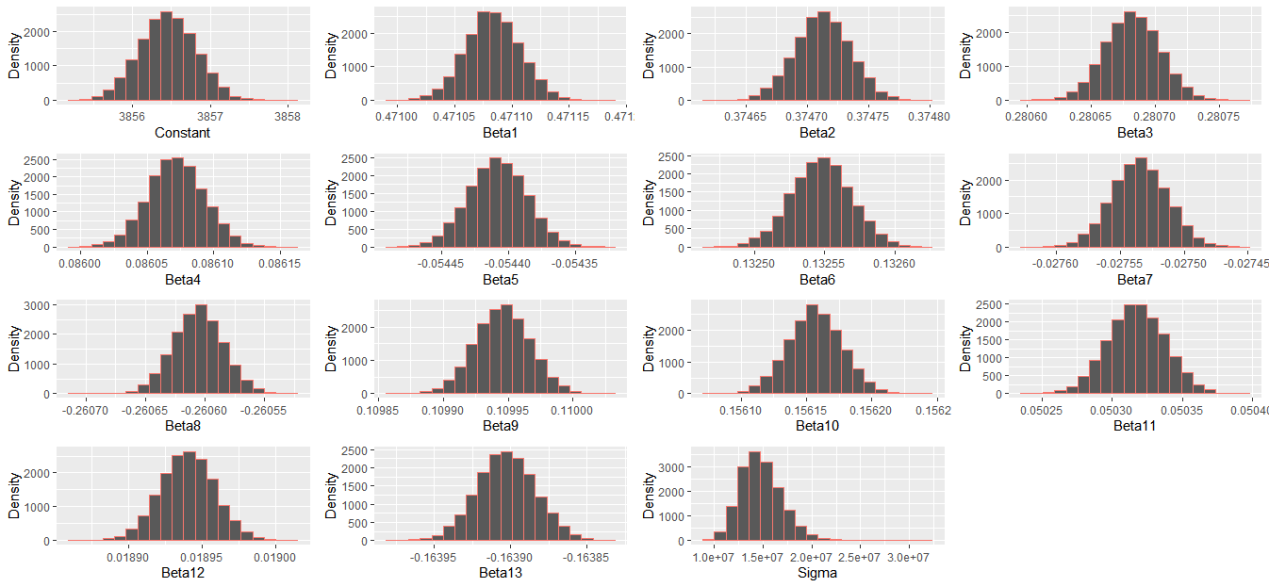


1. H4


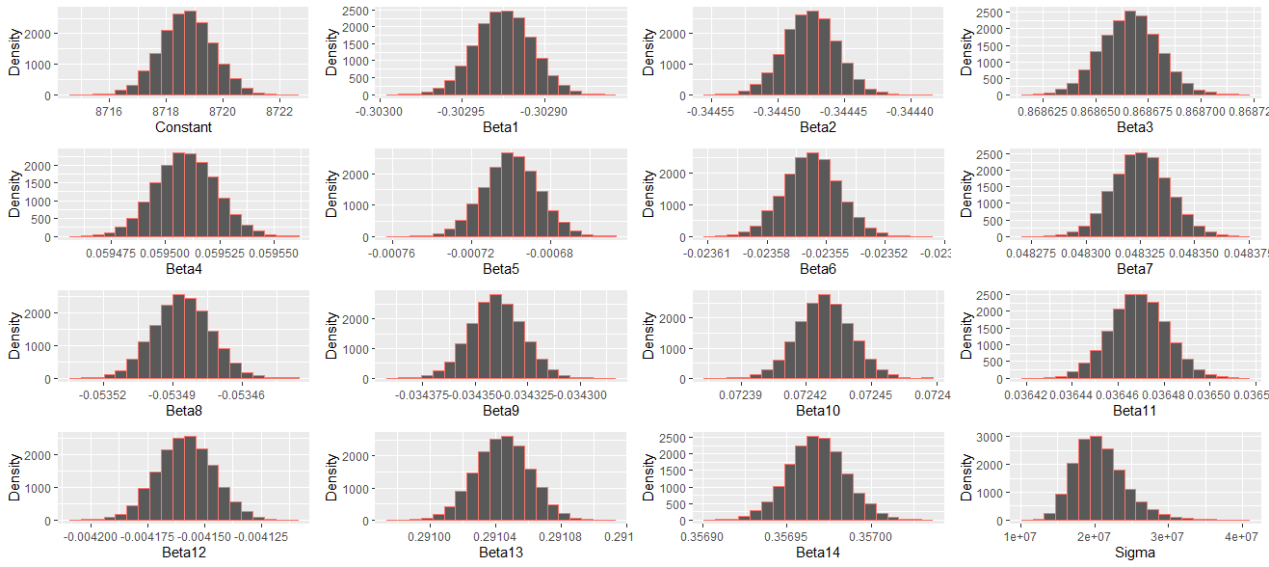


1. H5


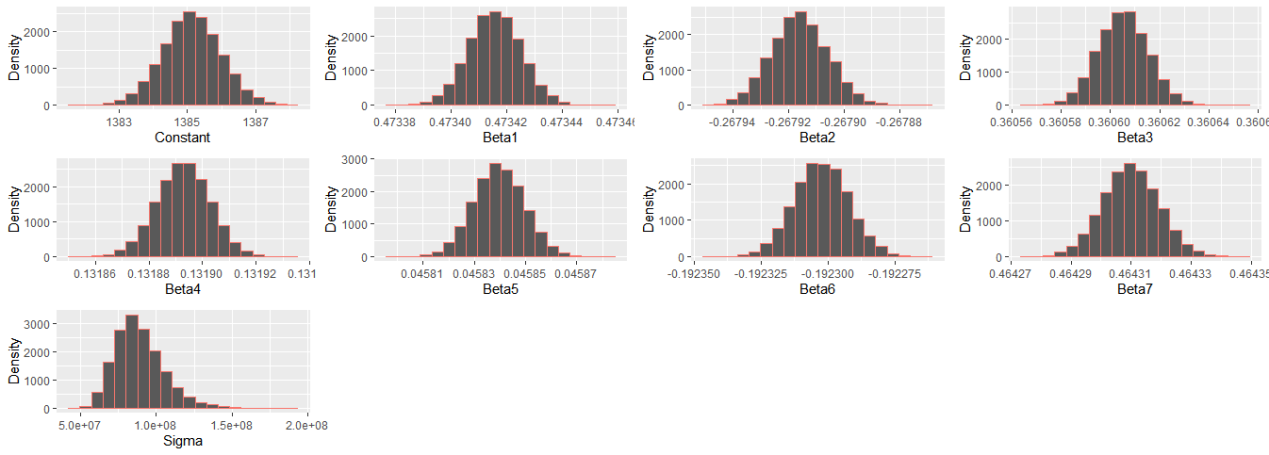


1. H6


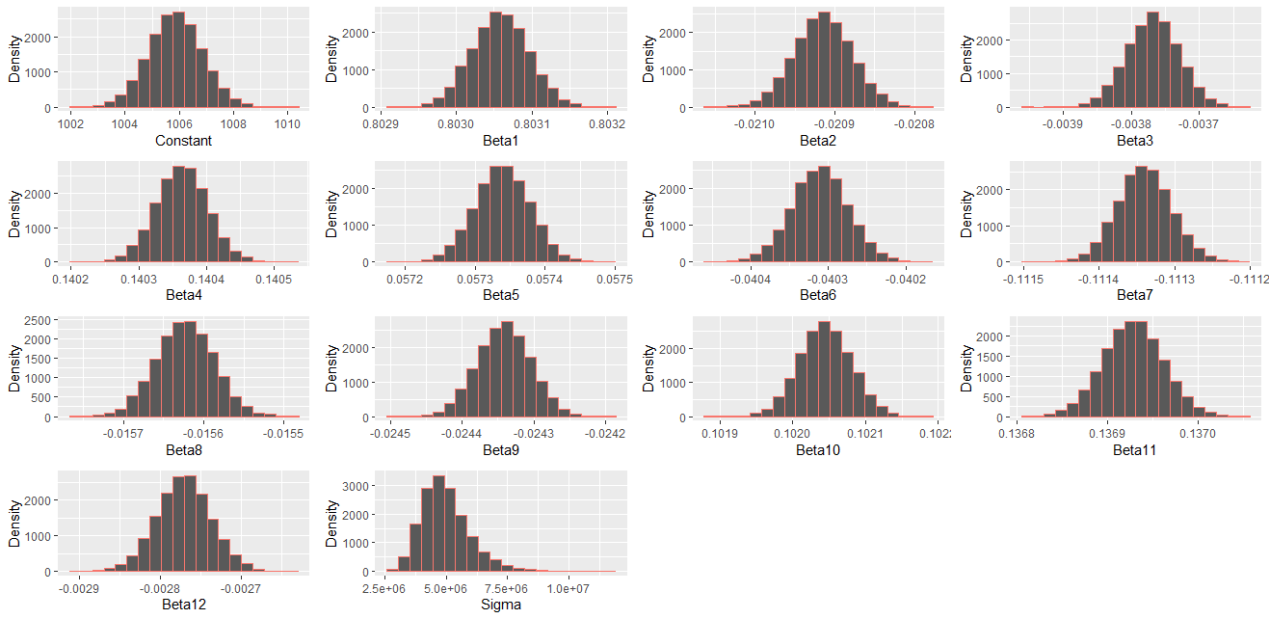


1. H7

Figure S 1: Posterior distribution of parameters of different models.

| 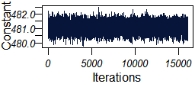 | 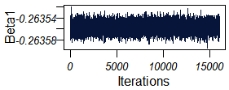 | 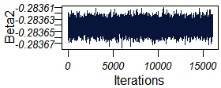 |
| --- | --- | --- |
| 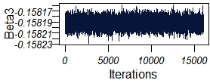 | 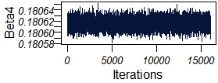 | 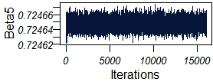 |
| 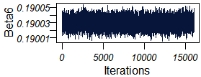 | 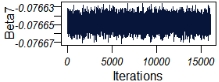 | 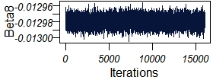 |
| 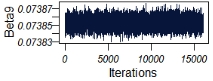 | 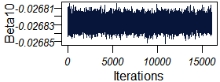 | 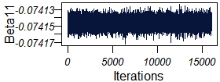 |
| 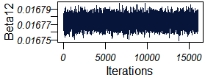 | 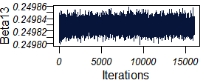 | 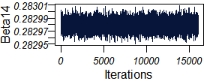 |
| 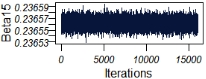 | 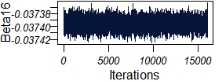 | 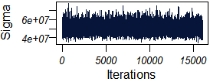 |
| (a) H1 | | |
| 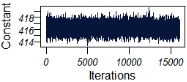 | 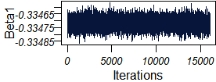 | 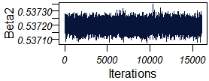 |
| 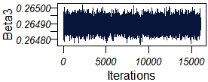 | 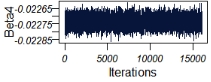 | 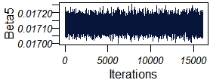 |
| 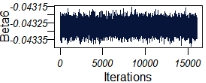 | 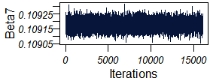 | 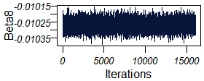 |
| 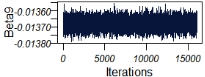 | 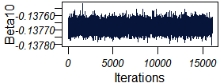 | 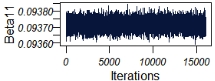 |
| 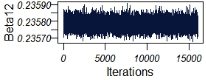 | 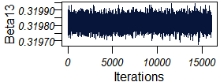 | 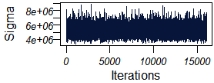 |
| (b) H2 | | |
| 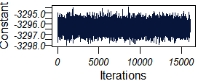 | 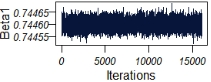 | 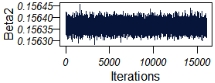 |
| 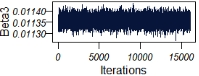 | 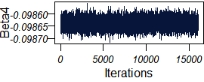 | 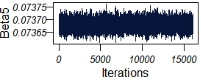 |
| 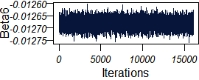 | 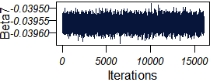 | 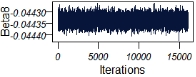 |
| 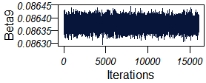 | 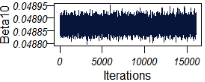 | 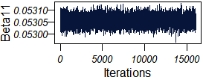 |
| 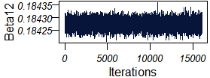 | 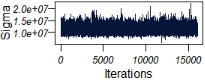 |  |
| (c) H3 | | |
| 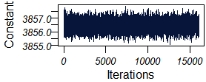 | 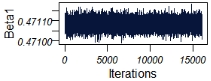 | 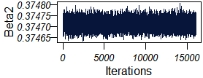 |
| 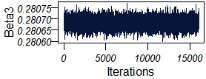 | 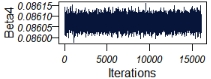 | 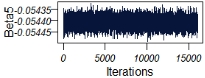 |
| 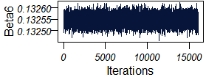 | 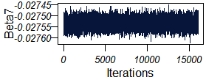 | 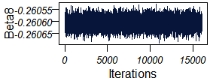 |
| 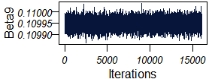 | 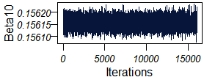 | 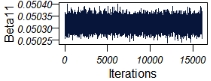 |
| 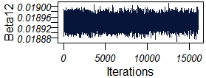 | 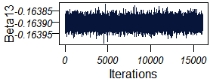 | 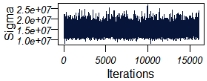 |
| (d) H4 | | |
| 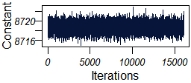 | 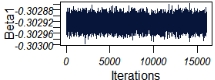 | 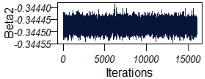 |
| 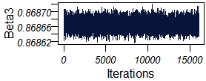 | 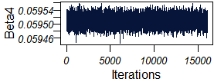 | 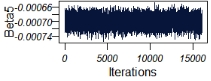 |
| 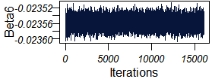 | 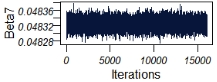 | 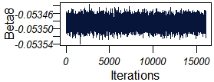 |
| 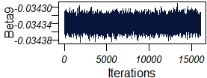 | 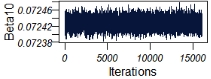 | 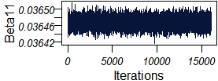 |
| 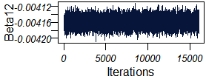 | 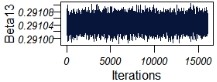 | 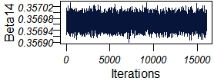 |
| 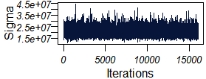 |  |  |
| (e) H5 | | |
| 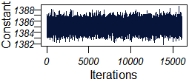 | 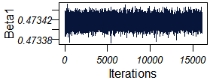 | 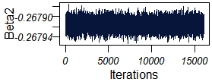 |
| 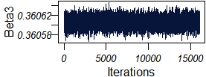 | 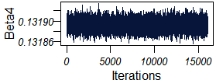 | 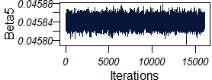 |
| 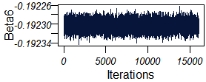 | 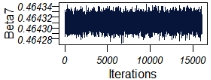 | 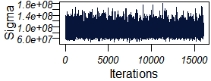 |
| (f) H6 | | |
| 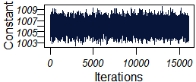 | 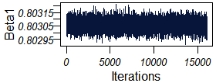 | 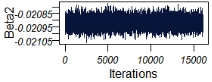 |
| 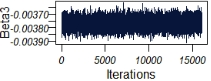 | 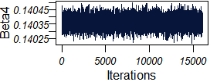 | 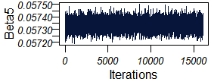 |
|  |  |  |
|  |  |  |
|  |  |  |
| (g) H7 | | |

Figure S 2: The trace plots of parameter simulation generated by Gibbs sampling algorithm.
